# Obsidian as a Raw Material for Eco-Friendly Synthesis of Magnetic Zeolites

**DOI:** 10.3390/ma13204633

**Published:** 2020-10-16

**Authors:** Claudia Belviso, Davide Peddis, Gaspare Varvaro, Maryam Abdolrahimi, Andrea Pietro Reverberi, Francesco Cavalcante

**Affiliations:** 1 Istituto di Metodologie per l’Analisi Ambientale—CNR, 85050 Tito Scalo, Italy; francesco.cavalcante@imaa.cnr.it; 2 Departments of Chemistry and Industrial Chemistry (DCIC), Università of Genova, 16146 Genova, Italy; davide.peddis@unige.it (D.P.); andrea.reverberi@unige.it (A.P.R.); 3 Istituto di Struttura della Materia—CNR, 00015 Monterotondo Scalo, Italy; abdolrahimi.ps66@yahoo.com; 4 Dipartimento di Scienze, Università degli Studi Roma Tre, 00146 Roma, Italy; gaspare.varvaro@ism.cnr.it

**Keywords:** obsidian, ultrasonic and hydrothermal water bath, zeolite, magnetic properties

## Abstract

A sample of rhyolitic obsidian (OS) was used as raw material for zeolite synthesis by long (4 days) and fast (2 h)-aging hydrothermal processes. Zeolite synthesis was also performed by a fast (2 h) sonication method. The products were analysed by X-ray diffraction (XRD) and scanning electron microscopy (SEM) both immediately after and 3 years after their formation in order to determine the stability of synthetic materials according to the method used. The results confirm zeolitization of obsidian both by long-aging conventional hydrothermal heating and fast hydrothermal process. However, the data highlight the efficiency of direct ultrasound energy in achieving more stable zeolite crystals over time. These results carried out using a natural source, follow those already obtained using wastes and pure sources as raw materials thus providing a definitive validation of the different mechanisms controlling zeolite formation according to the process used. Moreover, the results confirm the effectiveness of ultrasonic energy in the formation of zeolites that are more stable over time. Due to the chemical composition of the obsidian precursor, all synthetic zeolites show good magnetic properties (i.e., saturation magnetization), in view to potential magnetic separation.

## 1. Introduction

Zeolites are hydrated aluminosilicate minerals consisting of TO_4_ tetrahedra (T = Si or Al) which are connected by sharing corner oxygen atoms. They are characterized by peculiar and well-known proprieties (i.e., high surface area, porosity, and cation exchange capacity) making these minerals very useful in many applications [1,2,3,4,5,6,7,8,9]. However, with the aim to improve the zeolite use in some specific contexts (e.g., water pollution remediation), recent studies have been focused on the development of magnetic zeolites. Literature data have documented different methods to form magnetic zeolites, mainly based on addition of preformed iron oxide nanoparticles during the synthesis [10,11,12,13]. As an alternative we recently demonstrated that many kinds of magnetic zeolites can be obtained exploiting chemical composition of the precursors without the addition of external magnetic nanoparticles [14,15].

Due to the general growing demand for these materials, significant research activities have been devoted to zeolite synthesis also considering the economical stand, the environmental and safety implications for sustainable production [16]. As result, literature data have shown zeolite formation by different methods [17,18,19,20,21,22,23,24,25,26,27,28], mainly using processes at low aging temperatures [29,30,31,32,33,34]. In our previous manuscripts [35,36], the zeolite formation by conventional hydrothermal process or ultrasonic was analysed. The experiments were performed using two different raw materials based on silica and aluminum. In both cases, the results indicated that the hydrothermal process is responsible for slower geopolymer transformation into well-defined zeolite crystals, whereas the fast precipitation mechanism determines the formation of metastable zeolites by sonication. The data showed that the two different approaches also control the stability of the synthetic products over the years [35,36].

Beside the processes, the use of different precursors has also been extensively explored. Zeolites have been formed from wastes [37,38,39,40,41,42,43,44,45,46], natural sources such as clay minerals [47,48,49,50,51,52,53,54] or by adding zeolite seeds in starting gel with or without organic templates [55,56,57,58]. Recently, natural cost-effective and green silica precursors such as diatomite and obsidian were also used [59,60,61,62,63,64,65,66]. Both materials are interesting natural precursors due to their relatively low cost and highly reactivity although the formation of zeolite type strongly depends on the elemental composition of these natural sources as well as on the used process. Kawano and Tomita [65] demonstrated that phillipsite and merlinoite formed from alteration of obsidian in NaOH and KOH solution, respectively whereas Mamedova [64] formed natrolite with high degree of crystallization by a hydrothermal method at 200 °C using a mixture of natural halloysite and obsidian. In our previous paper [62], small crystals of EMT-type zeolite were synthesized from an obsidian precursor. The data displayed that zeolite formed at a lower incubation temperature using seawater comparing to distilled water. However, with seawater, EMT showed higher metastable behavior as indicated by its competitive growth with P-type zeolite [62].

In the present study, the efficiency of ultrasonic versus hydrothermal water bath method to convert obsidian into zeolite was investigated. Moreover, the stability over the time of the synthetic products formed from this natural precursor will be studied. Finally, due to the potential complex magnetic and microstructural properties of the obsidian [67], magnetic characterization of the precursor and final products were performed.

## 2. Materials and Methods

A sample of ryholitic obsidian (OS) collected at Punta delle Rocche Rosse (Lipari, Aeolian Islands, Italy), was used as raw material. It was pre-fused at 600 °C for 1 h with NaOH (1:1.2 weight ratio) and stirred for a night in a seawater solution. In detail, 8 g of pre-fused OS powder was mixed with 42 mL of seawater. Finally, the sample was submitted to three different aging processes in separate experiments: (i) a conventional hydrothermal process at 60 °C for 4 days (HY4d) [29,30]; (ii) a hydrothermal water bath method for 2 h at 60 °C (HY2h) [35,36]; (iii) an ultrasonic water bath process (240 W, 35 kHz) for 2 h at 60 °C (US2h) [35,36]. After all the aging processes, the solids and solutions were separated by centrifugation. The solids of all the samples were washed with the same amount of distilled water and dried in an oven at 80 °C. The mineralogical characterization of both OS and synthetic products (HY4d, HY2h US2h) were carried out by X-ray powder diffraction (XRD) using a Rint Miniflex powder diffractometer (Rigaku) with Cu-Kα radiation. Morphological observations were performed by scanning electron microscopy (SEM, Supra 40, Zeiss,) equipped with an energy dispersive spectrometer (EDS). Chemical composition of obsidian was determined for its major elements by X-ray fluorescence (XRF, PW 1480, Philips).

DC magnetization measurements were investigated using MPMS XL-5 (H_max_ ± 5T, Quantum Design) and VSM (H_max_ ± 2T, Microsense) magnetometers. Each sample, in the form of powder dispersed within epoxy resin, is placed inside the capsules of polycarbonate. The epoxy resin is used to avoid any powder movement during the measurements.

## 3. Results

### 3.1. Raw Materials

**Figure 1:**
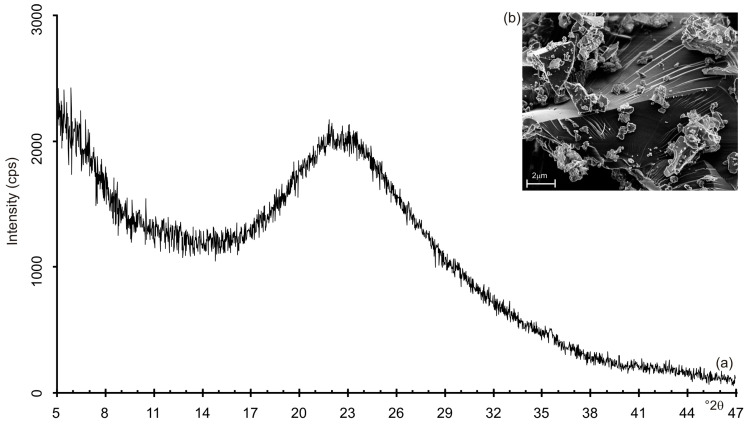
(**a**) X-ray pattern and (**b**) SEM picture of rhyolitic obsidian.

Figure 1 shows the XRD pattern and SEM images of the obsidian raw material. The X-ray profile shows the typical broad band of a glass material (Figure 1a) confirmed by the irregular particle morphology displayed by the scanning electron micrographs in Figure 1b. The chemical data (Table 1) indicate that obsidian is characterized by a high amount of SiO_2_ (75.48%) and relative low percentage of Al_2_O_3_ (11.75%) with a following SiO_2_/Al_2_O_3_ ratio of 6.42. The percentage of K_2_O is 5.41%, whereas the amounts of Na_2_O and Fe_2_O_3_ are 3.47% and 2.87%, respectively.

Finally, Table 2 shows the chemical composition and salinity of the natural seawater sample used. The data indicate a composition comparable with the typical average values of seawater.

### 3.2. Starting Process of Obsidian Zeolitization

**Figure 2:**
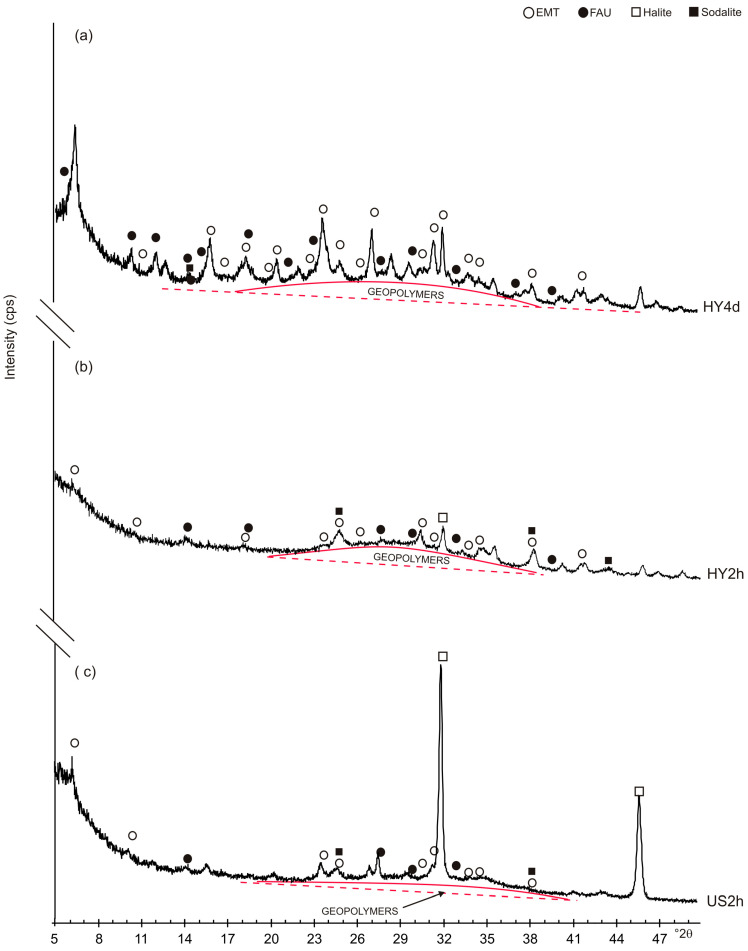
XRD patterns of the sample after: (**a**) conventional hydrothermal process at 60 °C for 4 days (HY4d); (**b**) hydrothermal water bath method for 2 h at 60 °C (HY2h) and (**c**) ultrasonic water bath (240 W; 35 kHz) for 2 h at 60 °C (US2h).

**Figure 3:**
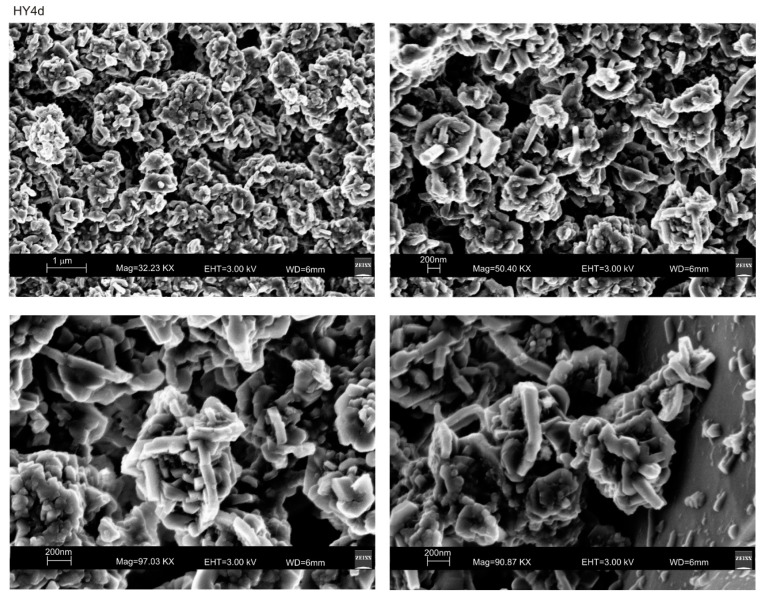
SEM images of EMT-type zeolite formed by 4-days conventional hydrothermal process (HY4d).

Figure 2 shows X-ray diffraction data after both HY and US processes. The XRD pattern of HY4d (Figure 2a) indicates the dominant presence of EMT-type zeolite whose typical hexagonal plate shape is shown by the SEM pictures in Figure 3. However, it cannot be excluded the presence of low amount of a faujasite type zeolite (FAU) phase and geopolymers as indicated by the broad band from 20 to 37° 2θ on the X-ray profile. XRD pattern of the obsidian sample incubated by the hydrothermal water bath method for 2 h (Figure 2b) indicates the presence of low amount of EMT-type zeolite confirming the presence of geopolymeric materials. Low percentages of sodalite and halide are also detectable. The presence of EMT-type zeolite is more evident in US2h XRD profile after ultrasonic water bath process for 2 h (Figure 2c). In this sample the amount of halite is quite high.

**Figure 4:**
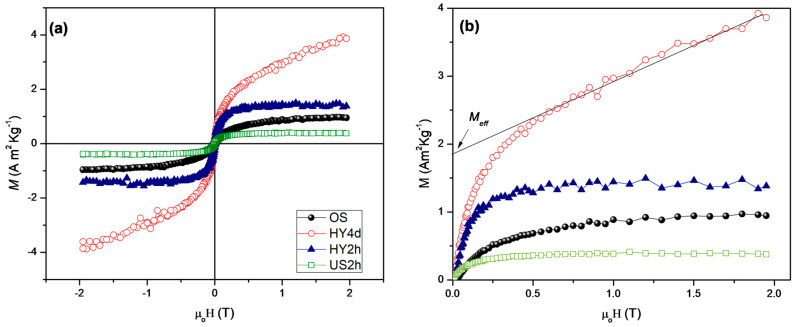
(**a**) Field dependence of magnetization recorded at 300 K; (**b**) Magnification M vs. H in the range 0–2 T. For all samples, extrapolation to zero of the high field linear portion of the magnetisation was used to determine the effective magnetic moment (M_eff_).

The field dependence of the magnetization of the OS raw material and all the synthetic zeolite samples has been investigated at 300 K (Figure 4a). The OS sample shows a ferromagnetic-like behavior with a very low saturation magnetization (M_s_ ≅ 0.96 A m^2^ kg^−1^), in agreement with the values found by other authors for obsidian samples [67].

This indicates that the magnetic behavior is dominated by some ferrimagnetic oxides like maghemite (γ-Fe_2_O_3_) or magnetite (Fe_3_O_4_) present in quite low percentages, in agreement with the chemical analysis. Both HY2d and US2h show also ferromagnetic-like behavior with M_s_ values of ~1.32 and ~0.43 A m^2^ kg^−1^ respectively. HY2d shows an increase of 27% of M_s_ with respect to the OS raw material, indicating that the hydrothermal treatment promotes the formation and stabilization of ferro(ferrimagnetic) oxides. On the other hand, ultrasound treatment (US2h sample) brings about a strong reduction (~56%) of M_s_ with respect to OS sample, inducing us to believe that a higher fraction of antiferromagnetic oxides (e.g., hematite, α Fe_2_O_3_) and hydroxides (e.g., goethite, FeOOH) is present [68].

This landscape drastically changes in the samples HY4d, showing an antiferromagnetic behavior superimposed to a small ferromagnetic contribution in the lower field region [15]. To quantify the effective magnetic moment (M_eff_) due to the ferro(ferri)magnetic component, the extrapolation to zero of the high-field linear portion can be used (Figure 4b) [15]. M_eff_ value is around 1.85 A m^2^ kg^−1^, that is much higher what is expected for nanostructured hematite (<0.5 A m^2^ kg^−1^) indicating that the ferromagnetic contribution could be ascribed to the presence of ferro (ferri)magnetic oxides and not to uncompensated spin of nanostructured hematite.

### 3.3. Synthetic Products over the Time

**Figure 5:**
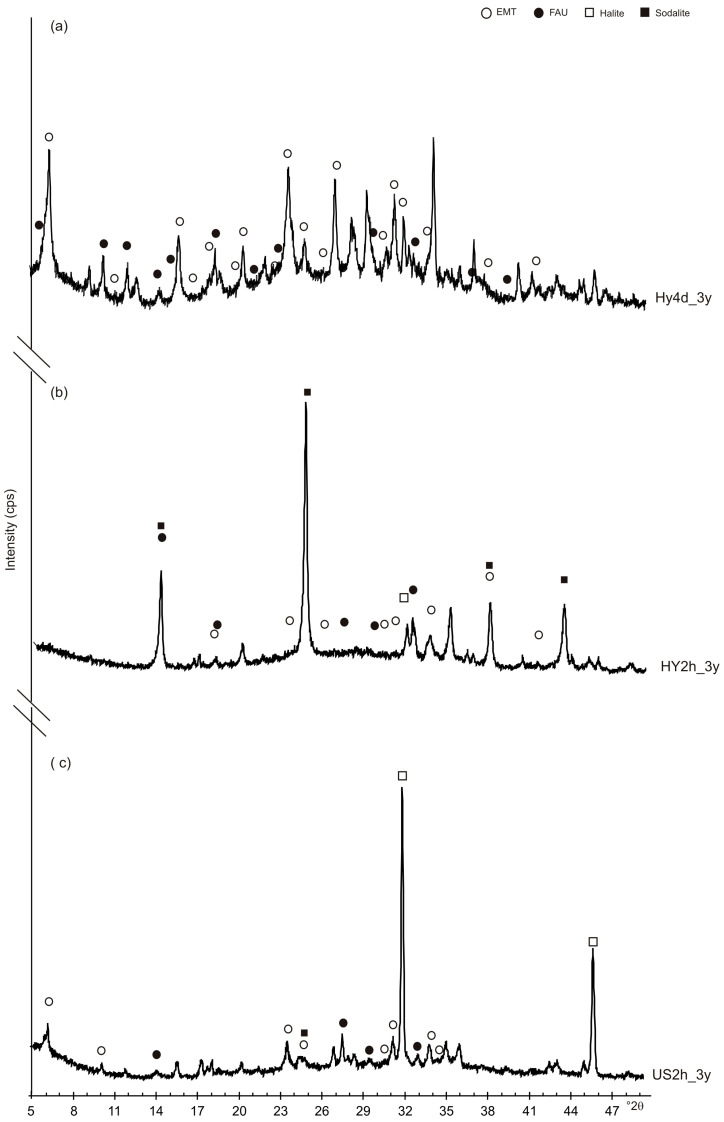
XRD profiles of the samples three years after their synthesis: (**a**) HY4d_3Y; (**b**) HY2h_3y and (**c**) US2h_3y.

In order to determine the stability of the synthetic products over the time, all the synthetic products were analysed again by XRD and SEM three years after their formation. Figure 5 shows XRD pattern of HY4d_3y, HY2d_3y and US2h_3y. The data indicate that the zeolites formed after 4 days of a conventional hydrothermal process and by the ultrasonic water bath process for 2 h are characterized by relatively stable behavior. In detail, XRD pattern of HY4d_3y (Figure 5a) indicates the presence of EMT-type zeolite combined with a lower amount of FAU. However, the peaks of sodalite seem to be higher comparing to the starting products (HY4d) (Figure 2a). The sample formed by a fast ultrasonic treatment (US2h_3y, Figure 5c) also shows a quite stable behavior three years after the zeolite formation (see Figure 2c), whereas a clear phase transformation takes place over the three years in the zeolite formed by the fast hydrothermal water bath method (HY2h). The X-ray profile in Figure 5b, in fact, displays the presence of sodalite as the main crystalline phase whereas the prepared sample is characterized by the presence of large amount of geopolymers and by low percentages of both EMT and FAU, together with low sodalite (Figure 2b).

## 4. Discussion

The results of this study can be considered as conclusive data confirming the role of different processes in controlling both the mechanism of zeolite formation and the stability of synthetic products over the time, regardless of the raw material used.

Our previous data already demonstrated that obsidian can be successfully used as a precursor material for the organic-template-free EMT-type zeolite synthesis by a conventional hydrothermal process at 60 °C using a seawater solution [62]. Similarly, in other previous works we showed that different crystallization mechanisms control the zeolite synthesis according to the processes used for their formation [35,36]. In those papers, a waste material (fly ash) and Na_2_O-Al_2_O_3_-SiO_2_-H_2_O precursor system were used to perform the experiments.

In the present study, we used one of the last types of natural raw material which remains to be investigated as a precursor to compare the efficiency of fast hydrothermal and ultrasonic water bath methods (2 h of treatment) in both type and zeolite stability over the time. Moreover, we also investigated the differences in both mechanism and zeolite stability between conventional long (4 days) and fast-aging hydrothermal processes (2 h).

The results indicate that all the synthetic products are characterized by the presence of EMT-type zeolite as dominant crystalline phase. However, HY4d and HY2h also show the presence of larger amount of geopolymers comparing to the US2h. The sonicated sample, instead, is characterized by higher amount of halite.

The differences between the investigated samples can be explained considering the crystallization mechanism related to the methods used. In our hypothesis EMT and FAU formation by hydrothermal process is controlled by a double-step mechanism characterized by geopolymer precipitation from saturated solution followed by a slow zeolite growth involving the amorphous mass. This hypothesis is in accordance with the results obtained using both waste sources [35] and pure reagents [36]. It also explains the presence of a larger amount of geopolymers in HY4d and HY2h as well as the behavior of these samples over time. The results, in fact, indicate that three years after their formation, HY2h_3y shows the presence of sodalite as its main phase (Figure 5b), thus confirming that the sample formed by fast hydrothermal water-bath method changed its mineralogical composition transforming the metastable EMT and FAU into a more stable form [69] through a progressive action involving the geopolymer phase.

However, the obsidian sample treated by a long-aging hydrothermal process does not show the same behavior. Three years after its formation, HY4d_3y, as opposed to HY2h_3y, is not characterized by the sole presence of sodalite. Its XRD pattern, in fact, shows a mineralogical composition qualitatively comparable with the starting one. This data suggests that the 4 days-aging hydrothermal process allows a more complete development of the double-step process for the zeolite formation, thus ensuring that the crystallization mechanism takes place slowly and therefore affording greater stability over time to the newly formed minerals. On the other hand, stopping this mechanism just two hours after activation (i.e., HY2h sample), determines the formation of EMT and FAU seeds with a stronger metastable behavior particularly displayed in a relative faster transformation at solid-state over time.

The presence of a high amount of halite and the stability over time of the samples formed by the fast ultrasonic water bath method indicate that the crystallization mechanism by sonication is controlled by a precipitation process, as already demonstrated in our previous papers [35,36]. The action of sonication improves both Na, Al, Si and Cl saturation and causes the disruption of nuclei already laid down in the medium, thus increasing the number of nuclei that quickly precipitate to form EMT and FAU-type zeolite together with halite.

Moreover, the magnetic results indicate that OS, HY2h and US2h show a ferromagnetic-like behavior while the fast hydrothermal and ultrasonic water bath treatment affect the saturation magnetization of the OS raw material. On the other hand, the long aging hydrothermal process dramatically changed the magnetic behavior, showing an antiferromagnetic-like behavior with a weak ferromagnetic contribution. The effective magnetic moment indicates that the ferromagnetic contribution could be due to the presence of ferro(ferri)magnetic oxides.

## 5. Concluding Remarks

The data indicate that obsidian can be converted into zeolitic material, mainly EMT-type zeolites exhibiting magnetic properties, by applying three different methods at low temperature (60 °C) and using a seawater solution. The synthesis was performed using fast hydrothermal or ultrasonic water bath methods (2 h of treatment) or a long-aging conventional hydrothermal process (4 days of treatment). The results confirm our previous data [35,36] indicating two different mechanisms for the zeolite synthesis represented by the direct fast zeolite precipitation using ultrasonic method and by geopolymer formation with a subsequent slower zeolite growth within the amorphous mass applying the hydrothermal process. Moreover, the data confirmed that the precipitation mechanism control of zeolite formation by sonication is also responsible for the stability of the synthetic products over time. However, a stronger antiferromagnetic contribution is observed after the longer hydrothermal prosses. The transformation of geopolymers into well-organized crystals characterizing the mechanism of zeolite formation by hydrothermal process is responsible for a slow but progressive transformation of metastable EMT and FAU-type zeolite into sodalite also using obsidian as raw material. However, the data indicate that this transformation into more stable forms is as fast as the time of hydrothermal water bath time is shorter.

## Figures and Tables

**Table 1:** Chemical composition of obsidian raw material.

*Major Constituents (wt.%)*											
**Sample**	**Na_2_O**	**MgO**	**Al_2_O_3_**	**SiO_2_**	**P_2_O_5_**	**K_2_O**	**CaO**	**TiO_2_**	**MnO**	**Fe_2_O_3_**	**SiO_2_/Al_2_O_3_**
OS	3.47	0.00	11.75	75.48	0.02	5.41	0.81	0.11	0.10	2.87	6.42
*Trace elements (ppm)*											
**Sample**	**Ni**	**Co**	**Sr**	**Zr**	**Cu**	**Zn**	**As**	**Rb**	**Sn**	**Cs**	**Pb**
OS	9.75	2.25	17.00	191.00	4.67	60.01	33.00	312.00	30.00	23.00	27.00

**Table 2:** Seawater composition (values in g/L; salinity g/kg).

Sample	Salinity	Si	Al	Cl	Na	Mg	Ca	K
Natural seawater	35.00	0.0015	0.0011	19.962	7.727	1.375	0.538	0.014
